# Activating Killer-cell Immunoglobulin-like Receptor genes confer risk for Crohn’s disease in children and adults of the Western European descent: Findings based on case-control studies

**DOI:** 10.1371/journal.pone.0217767

**Published:** 2019-06-13

**Authors:** Suzanne Samarani, David R. Mack, Charles N. Bernstein, Alexandre Iannello, Olfa Debbeche, Prevost Jantchou, Christophe Faure, Colette Deslandres, Devendra K. Amre, Ali Ahmad

**Affiliations:** 1 Laboratory of Innate Immunity, CHU Sainte-Justine Research Center/Department of Microbiology, Infectious Diseases & Immunology, University of Montreal, Montreal, Quebec, Canada; 2 Department of Gastroenterology, Hepatology & Nutrition, Children’s Hospital of Eastern Ontario, Ottawa, Ontario, Canada; 3 IBD Clinical & Research Center, University of Manitoba, Winnipeg, Manitoba, Canada; 4 CHU Sainte-Justine Research Center/Department of Pediatrics, University of Montreal, Montreal, Quebec, Canada; Centro Cardiologico Monzino, ITALY

## Abstract

**Background:**

Killer-cell Immunoglobulin-like Receptor (KIR) genes encode receptors, which are mainly expressed on, and control functional activities of, Natural Killer (NK) cells. There exist six distinct activating KIR genes in humans, who differ from one another with respect to the repertoire of these genes. Because activated NK cells can potentially cause tissue destruction, we hypothesized that variation in the inherited activating KIR genes in humans is associated with their innate susceptibility/resistance to developing Crohn disease (CD).

**Methods:**

We performed case control studies on three independent Canadian CD patient cohorts (all of the Western European descent): two comprising children (Montreal having 193 cases and 245 controls, and Ottawa having 93 cases and 120 controls) and the third one comprising predominantly adults (Winnipeg having 164 cases and 200 controls). We genotyped cases and controls for activating KIR genes by PCR with gene-specific primers and investigated associations between the genes and cases using unconditional logistic regression.

**Results:**

We observed strong associations between all the six KIR genes and CD in Ottawa children, with the strongest risk observed for the KIR2DS1 (p = 1.7 x10^-10^). Associations between all but the KIR2DS2 were replicated in the Montreal cohort with the strongest association evident for the KIR2DS5 (8.0 x 10^−10^). Similarly associations between five genes were observed in the adult Winnipeg cohort. In this cohort, strongest associations were evident with the KIR2DS5 (8.75 x 10^−8^). An overall analysis for all cohorts showed strong associations with four of the genes, with the strongest association evident for the KIR2DS5 (p = 1.35 x 10−^17^). In the combined analysis for four KIR genes, individuals carrying one or more of the KIR genes were at significantly higher risks for acquiring CD (p = 3.5 x 10^−34^).

**Conclusions:**

Activating KIR genes are associated with risk for developing CD in both children and adults.

## Introduction

Crohn’s Disease (CD), a type of inflammatory bowel disease (IBD), is a chronic, relapsing and remitting disease of the gastrointestinal tract. Considered a disease commonly affecting children and young adults, it is one of the most prevalent gastrointestinal tract diseases in the industrialized world. In North America alone, it affects one in three hundred children and its incidence appears to be on the rise in the developing countries; reviewed in [1,2]. As no cure for the disease exists, morbidity from the disease is quite high in particular in children who suffer from numerous consequences such as frequent surgery, growth impairment and psychosocial distress [2,3]. Deciphering the etio-pathogenesis of the disease is the focus of ongoing research.

Although the exact pathogenesis of CD remains unclear, it is believed to result from an excessive immune response of the host to intestinal microbiota, food constituents and/or self-antigens [4,5]. Several twin and family-based epidemiological studies have shown that genetic predisposition plays an important role in the development of this disease [6]; reviewed in [7]. Therefore, it is not surprising that a major research effort has been directed towards identification of genetic determinants that predispose humans to develop this disease. Genome-wide association studies (GWAS) as well as their meta-analyses have led to identification of ~150 susceptibility loci/genes for CD [8–12]. Among the strongest genetic determinants associated with the development of this disease include those that encode interleukin (IL)-23 receptor, Nucleotide-binding and Oligomerization Domain-containing protein (NOD)-2, the Autophagy-16-like Protein-1 (ATG16L1) and the Immunity-related GTPase M (IRGM) [13–16]. These discoveries have increased our understanding about the immunopathogenesis of the disease. Collectively, results from these studies strongly suggest the implication of innate immunity pathways in the development of this disease. Currently identified genetic determinants, however, account for ~10–20% of the inherited variability in CD [17]. Therefore, research for exploring novel genetic determinants of the disease still remains an area of priority.

Although GWAS have identified key genetic determinants of CD, coverage of current chips is limited to the areas of the genome known to harbour single-nucleotide polymorphisms (SNPs) and some small insertion-deletions. A large portion of the genome is characterized by structural variations that encompass copy-number variations, entire gene deletions or multiple genes of high homology. There is currently growing interest in understanding whether genetic determinants present in such regions of the genome could be associated with CD susceptibility. Interestingly, there are numerous portions of the genome that cannot be adequately tagged by SNPs implying that their direct study via a candidate gene approach would be necessary to identify potential susceptibility genes. One such region of the genome is the Leucocyte Receptor Complex (LRC) on chromosome 19q13.4, a dense region spanning ~150 kb that harbours many key genes that influence immune function [18,19]. Of particular interest are genes termed as the Killer-cell Immunoglobulin-like Receptor (*KIR*) genes located within the LRC that code for receptors, which are expressed on the surface of immune cells such as the Natural Killer (NK) cells and a subset of antigen-experienced CD8+ T lymphocytes (CTL), and whose expression regulates the functional activities of these cells. The *KIR* family comprises sixteen genes, which are located in a tandem head to tail arrangement [20]. Of these, seven *KIR* genes (*KIR2DL1*, *KIR2DL2/3*, *KIR2DL5A*, *KIR2DL5B* and *KIR3DL1-3*) encode receptors with long-tailed cytoplasmic tails. These receptors inhibit the functions of the immune cells upon stimulation with their cognate MHC class I ligands. Hence, these genes are called inhibitory *KIR* genes. Six of the *KIR* genes (*KIR2DS1-5* and *KIR3DS1*) are called activating genes, as they encode short-tailed receptors that activate the immune cells upon binding with their cognate ligands. In addition to gene polymorphism, KIR haplotypes also vary from one another with respect to the number of activating *KIR* genes. Some haplotypes, named as Group A KIR haplotypes, often carry only one activating *KIR* gene, *KIR2DS4* [21]. This gene is often mutated due to the presence of a 22 base pair deletion in exon 5. The deleted variant encodes a protein with only Ig-like domain and is non-functional, as it cannot be expressed on the cell surface as a receptor [22,23]. On the other hand, group B haplotypes often carry a full complement or a subset of any six activating *KIR* genes [21]. Consequently, humans differ from one another with respect to the number of inherited activating *KIR* genes. The inheritance of activating *KIR* genes is thought to increase immune competence of the individual. Such individuals are likely to clear viral infections and be less susceptible for malignancy. However, they are also likely to be more susceptible to autoimmune and chronic inflammatory diseases. Indeed several activating *KIR* genes have been associated with protection from infectious diseases like AIDS, HCV-induced hepatitis, etc as well as with susceptibility to certain autoimmune and chronic inflammatory diseases like Type 1 diabetes, leukemia and different forms of arthritis [24–31]; reviewed in [32]. Few studies have investigated the potential association of activating *KIR* genes with CD. We addressed this issue and report here that in Canadians of the Caucasian ancestry, activating *KIR* genes confer strong susceptibility to the development of this disease.

## Materials & methods

### Patient populations and DNA extraction

We carried out a case-control study based on cohorts acquired from three gastroenterology clinics at Ottawa, Montreal, and Winnipeg, in Canada. The Ottawa cohort comprised unrelated cases and controls of white (Western European) origin (cases = 93, controls = 120), the Montreal cohort comprised of Caucasian unrelated cases and controls that were exclusively of French ancestry (cases = 193, controls = 245), and the Winnipeg cohort comprised unrelated predominantly adult cases and controls of Caucasian origin (cases = 164, controls = 200). The diagnosis of the CD cases was based on standard criteria that included clinical, radiological, endoscopic and histopathological findings. As *KIR* genes frequencies may vary between apparently ethnically homogeneous populations, we selected controls from different sources to enhance population representation. These included children visiting the orthopaedic department of the study hospital for minor fractures (Ottawa and Montreal), their siblings (Montreal), a random sample of population-based children and adults (Montreal and Winnipeg) and a birth cohort (Montreal). Only healthy controls without any cancer or autoimmune disorders were included. A subset of the control subjects from Ottawa and Montreal have been previously utilized for replicating or confirming associations between reported susceptibility genes (viz. *NOD2*, *ATG16L1*, *IL23R*, *IL10*, *STAT3*, *ZNF365*, *PTPN2*, locus 20q13 etc.) and CD in Canadian children [33–37]. Blood and/or saliva samples were collected from the participants as a source of DNA. Genomic DNA from the samples was extracted, quantified by UV spectrometry, coded and aliquoted. The aliquots were kept at -80˚C in the bank maintained at the CHU Sainte-Justine Research Center.

### Ethical statement

Informed written consent was obtained from each study participant or his/her legal guardian, and the Institutional Ethics committee, Comité d'éthique de la recherche CHU Sainte-Justine (CER CHUSJ), approved the studies.

### KIR genotyping

The presence or absence of each activating KIR in all cases and controls was determined by using PCR and sequence-specific primers on their genomic DNA samples as described [38–41]. Standard PCR protocols were used which comprised initial denaturation at 95˚C for 5 minutes followed by 30–35 cycles, each comprising 30 seconds of denaturation at 95˚C, 30 seconds of annealing at 60–68˚C and 2 minutes of extension at 72˚C. The final step included extension for 10 minutes. The optimal conditions for PCR (number of PCR cycles and annealing temperatures) were determined for each gene in preliminary experiments. The amplified DNAs from the PCR reactions were run on 2–2.5% agarose gels and electrophoresed in Tris-buffered saline (TBS) buffer. The gels were stained with ethidium bromide, scanned and imaged. The detection of a DNA band of the expected molecular weight was considered as the presence of the gene in the genomic DNA sample. In the case of *KIR2DS4*, two sets of primers were used to determine whether the gene was present, and if so, whether it had the 22 bp mutation in its exon 5. Since the *KIR2DS4* variant with the 22 bp deletion encodes a protein that cannot be expressed on the cell surface as a receptor, its presence was considered as absence of the gene [22,23]. We used DNA samples known to be positive or negative for the gene as positive and negative controls, respectively. The PCR reactions included a negative control (the reaction without DNA template) as a safeguard against false positive cases as well as a positive control for HLA-DRB, as a safeguard against false negative cases.

### Population stratification

We examined the presence of population stratification in the three cohorts by examining null markers (n = 26) across the genome. A subset of cases/controls from each of the 3 cohorts was genotyped and the inflation factor (lambda, λ) was estimated using the GC program [42].

### Statistical analysis

All study participants were genotyped with respect to the presence or absence of all six activating KIR genes. After assessing quality control (false positive, false negative, non-specific bands, contaminations, etc), we compared the KIR gene frequencies between cases and controls using Chi-square tests. Subsequently, we carried out unconditional logistic regression analyses, fitting a separate model for each activating KIR gene. We also assessed the effects of harbouring multiple activating KIR genes. For this purpose, we carried out an analysis considering the carriage of activating genes as a continuous variable. We examined homogeneity of noted associations for each gene using chi-square statistics. Genes showing homogeneous associations were further examined on the pooled cohorts using logistic regression. Odds ratios (OR), corresponding 95% confidence intervals (95% CI), and p-values were estimated. To account for multiple comparisons for six genes, p-values ≤ 1 x 10^−3^ were considered statistically significant. All analyses were carried out using STATA software (version 10.1; STATA Corp TX USA).

## Results

The clinical and demographic characteristics of the CD cases are shown in Table 1. Based on 26 null markers, we found no evidence of population stratification in any of the 3 cohorts (λ = 1.0, 1.02 and 1.0 respectively for the Ottawa, Montreal and Manitoba cohorts). As the laboratory methods utilized could not distinguish between those who had one or two genes, tests for Hardy-Weinberg could not be implemented. Initially we conducted this study in the Ottawa cohort of Canadian children of Caucasian ancestry. Initially we conducted this study in the Ottawa cohort of Canadian children of Caucasian ancestry. As shown in Table 2, the frequencies of most of the activating *KIR* genes were significantly higher (p≤0.001) in the cases than in the controls suggesting that the inheritance of these genes enhanced risk for developing CD. The gene that posed the maximum risk in this population was *KIR2DS1* (OR = 7.2, 95% CI = 3.78–13.7, p-value = 1.7 x 10^−10^). The gene rank in conferring the CD risk was *2DS1>2DS2 >2DS4>2DS3>2DS5>3DS1*.

**Table 1:** Clinical characteristics of the CD patient cohorts.

Characteristic	Ottawa	Montreal	Winnipeg
	N = 93	N = 193	N = 164
Age at diagnosis			
≤16	82 (88.2)	174 (90.2)	15 (9.1)
>16–40^†^	11 (11.8)	19 (9.8)	108 (65.9)
>40			41 (25.0)
Gender (%)			
Females	44 (47.3)	91 (47.2)	98 (59.8)
Males	49 (52.7)	102 (52.8)	66 (40.2)
Disease location (%)^†^			
L1±L4	23 (25.3)	38 (20.1)	77 (47.2)
L2±L4	15 (16.5)	64 (33.9)	38 (23.3)
L3±L4	53 (58.2)	87 (46.0)	48 (29.4)
Disease behaviour (%)^†^			
B1±p	71 (76.3)	172 (90.1)	87 (53.0)
B2±p	7 (7.5)	9 (4.7)	45 (27.4)
B3±p	15 (16.1)	10 (5.2)	32 (19.5)

^†^Classification based on the WGO’s Montreal Classification. The age at diagnosis cut-off for study inclusion was 16 years in Ottawa and 20 years in Montreal. Four patients had disease restricted to either only the upper tract or anal region and for three patients the location could not be adequately classified. For two patients, the disease behaviour at diagnosis was not clear. The percentages may not add up due to rounding.

**Table 2:** Association between the presence/absence of activating KIR genes and risk for CD in the Ottawa pediatric cohort.

Gene	Patients	Controls	OR (95% CI)	P-value
	N (%)	N (%)		
2DS1				
+	76 (81.7)	46 (38.3)	7.2 (3.78–13.7)	1.7 x 10^−10^
-	17 (18.3)	74 (61.7)		
2DS2				
+	78 (83.8)	75 (62.5)	3.12 (1.60–6.07)	0.001
-	15 (16.10	45 (37.5)		
2DS3				
+	47 (50.5)	34 (28.3)	2.58 (1.46–4.56)	0.001
-	46 (49.50)	86 (71.7)		
2DS4				
+	71 (76.3)	66 (55.0)	2.64 (1.45–4.80)	0.001
-*	22 (23.7)	54 (45.0)		
2DS5				
+	43 (46.2)	32 (26.7)	2.37 (1.33–4.20)	0.003
-	50 (53.8)	88 (73.3)		
3DS1				
+	54 (58.1)	47 (39.2)	2.15 (1.24–3.73)	0.006
-	39 (41.9)	73 (60.8)		

* indicates the presence of the 22 bp deleted forms and/or absence of the gene

We searched for potential replication in another independent and ethnically homogeneous population and investigated the presence or absence of the six activating *KIR* genes in the Montreal cohort (all of which comprised Canadian children of the French ancestry). As shown in Table 3, the results were almost similar to those obtained with the Ottawa cohort. All the activating *KIR* genes showed higher frequencies in the cases than in the controls. Associations with four *KIR* genes were strongly significant (p-values ranging from 10^−3^ to 10^−10^). Associations with *KIR2DS4* were marginally non-significant after correcting for multiple comparisons. No associations were evident for *KIR2DS2*. In the Montreal cohort, the carriage of *KIR2DS5* gene posed the maximum risk (OR = 3.58, 95% CI = 2.39–5.37, p = 8.0 x 10^−10^). The gene rank in conferring the CD risk was *2DS5>2DS1>3DS1>2DS3>2DS4*.

**Table 3:** Association between the presence/absence of activating KIR genes and risk for CD in the Montreal pediatric cohort.

Gene	Patients	Controls	OR (95% CI)	P-values
	N (%)	N (%)		
2DS1				
+	127 (65.8)	102 (41.6)	2.70 (1.8–4.0)	6.7 x 10^−7^
-	66 (34.2)	143 (58.4)		
2DS2				
+	123 (63.7)	138 (56.3)	1.36 (0.93–2.0)	0.12
-	70 (36.3)	107 (43.7)		
2DS3				
+	111 (57.5)	83 (33.9)	2.64 (1.79-.3.90)	1.01 x 10^−6^
-	82 (42.5)	162 (66.1)		
2DS4				
+	137 (71.0)	139 (56.7)	1.87 (1.25–2.78)	0.002
-*	56 (29.0)	106 (43.3)		
2DS5				
+	140 (72.5)	104 (42.4)	3.58 (2.39–5.37)	8.0 x 10^−10^
-	53 (27.5)	141 (57.6)		
3DS1				
+	126 (65.3)	101 (41.2)	2.68 (1.81–3.96)	7.4 x 10^−7^
-	67 (34.7)	144 (58.8)		

* indicates the presence of the 22 bp deleted forms and/or absence of the gene

To investigate whether noted associations with the KIR genes and CD were unique to children, we examined these associations in a predominantly adult Caucasian cohort (~90% cases >16 years of age at diagnosis) of CD from Winnipeg. As shown in Table 4, significant associations were evident for 5 of the 6 genes. The carriage of *KIR2DS5* gene posed the maximum risk (OR = 3.27, 95% CI = 2.09–5.12, p = 8.75 x 10^−8^). In all three cohorts, harbouring the functional *KIR2DS4*, which is the only activating *KIR* found in A haplotypes, enhanced risk for CD compared with harbouring no and/or the mutant gene.

**Table 4:** Association between the presence/absence of activating KIR genes and risk for CD in the adult Winnipeg cohort.

Gene	Patients	Controls	OR (95% CI)	P-value
	N (%)	N (%)		
2DS1				
+	91 (55.5)	94 (47.0)	1.41 (0.93–2.12)	0.11
-	73 (44.5)	106 (53.0)		
2DS2				
+	107 (65.2)	85 (42.5)	2.54 (1.66–3.89)	1.9 x 10^−5^
-	57 (34.8)	115 (57.5)		
2DS3				
+	98 (59.8)	67 (33.5)	2.95 (1.92-.4.52)	8.14 x 10^−7^
-	66 (40.2)	133 (66.5)		
2DS4				
+	115 (70.1)	104 (52.0)	2.17 (1.40–3.34)	5.0 x 10^−4^
-*	49 (29.9)	96 (48.0)		
2DS5				
+	122 (74.4)	94 (47.0)	3.27 (2.09–5.12)	8.75 x 10^−8^
-	42 (25.6)	106 (53.0)		
3DS1				
+	119 (72.6)	93 (46.5)	3.04 (1.96–4.73)	8.14 x 10^−7^
-	45 (27.4)	107 (53.5)		

* indicates the presence of the 22 bp deleted forms and/or absence of the gene. The analysis from 170 cases and 200 controls are shown.

Barring 2 genes (*2DS1* and *2DS2*) associations between other four activating *KIR* genes were homogenous across the cohorts. A combined analysis for these 4 genes showed strong associations between all the genes (Table 5) (p-values ranging from 10^−9^ to 10^−11^) and CD. As each *KIR* gene appeared to confer risk individually, we assessed whether carriage of multiple *KIR* genes further enhanced it in children (Table 6). Increasing carriage of KIR genes was strongly associated with risk for CD in all 3 cohorts individually as well as in the combined cohorts (p-value = 7.5 x 10^−35^ for carriage of >5 genes).

**Table 5:** Association between the presence/absence of 4 activating KIR genes and risk for CD in the pooled cohorts*.

Gene	OR (95% CI)	P-value
2DS3	2.73 (2.11–3.53)	8.0 x 10^−15^
2DS4	2.14 (1.64–2.78)	8.15 x 10^−9^
2DS5	3.06 (2.36–3.97)	1.35 x 10^−17^
3DS1	2.66 (2.06–3.44)	4.0 x 10^−14^

* Based on implementing the logistic regression model after accounting for study site.

**Table 6:** Association between number of activating KIR genes and CD in the children cohorts.

	Ottawa	Montreal	Pooled*
Number of genes	OR (95% CI)	OR (95% CI)	OR (95% CI)
	P-value	P-value	P-value
≤ 2	Reference	Reference	Reference
3–4	8.1 (3.84–17.1)	3.83 (2.3–6.38)	4.9 (3.21–7.47)
	2.0 x 10^−8^	2.2 x 10^−7^	1.3 x 10^−13^
5–6	12.9 (5.36–31.1)	13.4 (7.22–25.0)	13.5 (8.10–22.36)
	1.2 x 10^−8^	2.4 x 10^−16^	1.0 x 10^−23^
P-value for trend**	2.1 x 10^−9^	1.8 x 10^−16^	2.5 x 10^−24^

* Pooled estimates after accounting for study site.

** based on including the variable representing the number of KIR genes as a continuous variable in the logistic regression model

## Discussion

To the best of our knowledge, this is the first study implicating multiple activating *KIR* genes in enhancing the risk for developing CD both in children and adults of the white origin. It is noteworthy that the magnitudes of the associations noted here for most of the genes are comparable to the ones reported earlier for *NOD2* and *IL23R*, which represent the most strongly associated genes with CD [15,16]. So far, a few studies have investigated potential associations between *KIR* genes and susceptibility/resistance to developing CD. In one study, Wilson et al [43] examined associations between KIR genes and CD in a Brazilian white population comprising of 137 CD patients and 250 healthy unrelated individuals. Although no independent effects with either of the KIR genes were observed, some interactions between the KIR genes and their HLA ligand genes were noted. Jones et al [44] showed that the *KIR2DS2* gene was associated with enhanced risk for Ulcerative Colitis (the other less severe type of IBD) but not for CD in white adults (of the Western European descent). Similarly, Hollenbach et al [45] did not find any association of activating or inhibitory *KIR* genes with CD in a North American population. More recently, Saito et al [46] investigated *KIR* variability in a relatively small number (fifty) of Japanese CD patients and 325 healthy controls. They found no significant association of any *KIR* gene with the disease, however, the frequency of *KIR2DS3* was significantly increased and that of *KIR2DS4* was decreased in UC patients. In this regard, a recent meta-analysis of five published studies [47] reported a negative association of one *KIR* gene (*KIR2DS3*) with CD risk. Of relevance here is a published meta-analysis of GWAS by Jostins et al [4]. They identified several SNPs in significant associations with both CD and UC. The eQTL analysis showed that one of the SNPs, rs11672983, was associated with the expression of genes within the KIR gene complex. In line with this finding, Lopez-Hernandez et al. [48] identified KIR2DS1 and KIR2DS5 as significant risk factors in Spanish IBD patients. Taken together, the studies suggest that imbalances between activating and inhibitory KIR genes and their ligands may explain, at least in part, the pathogenesis of the inflammatory bowel diseases. Our results are in concordance with these conclusions and suggest a stronger role of activating KIR genes in conferring CD risk. Since the inhibitory *KIR-HLA* genotypes with different inhibitory potentials for NK cells (e.g., *KIR2L2/3-HLA-C1* vs *KIR2DL1-HLA-C2*) have been differentially associated with human diseases including CD [28,29,45,46], we are currently investigating whether inhibitory *KIR* genes and their cognate MHC class I alleles show any association with CD in our cohorts.

It is interesting that significant associations with activating *KIR* genes have not been previously demonstrated in GWAS studies. We believe the reason is the extreme homogeneity of the region that limits the use of high-throughput technologies in adequately capturing variation in the complex. A testament to this are the observations that even in the 1000 genome project, the sequencing technologies used have been unable to capture the region (null sequences reported). We thus believe that for regions of such homogeneity, manual PCR based methods (such as the one we utilized here) are most informative.

Unlike inhibitory KIR, most of which are known to bind specifically to a subset of MHC class I antigens, the specific ligands for activating KIR remain relatively undiscovered. In this regard, KIR2DS4 was shown to bind to certain MHC class I antigens but with relatively lower affinity [49]. In fact it was later shown to bind an unidentified non-MHC ligand expressed by primary human melanoma cells [50]. In regard, KIR2DS1 (as well as its inhibitory counterpart KIR2DL1) was recently shown to bind a specific peptide (SRGPVHHLL) in association with a group II HLA-C (HLA-C*06:02) [51]. Interestingly, KIR3DS1 was shown to bind open conformers of HLA-F, a non-classical MHC class I antigen [52]. Our results suggest that the intestinal epithelial cells from CD patients are likely to express ligands for activating KIR. Given that activating *KIR* genes have been shown to confer susceptibility/resistance towards human diseases, finding specific ligands for the encoded receptors should be an area of high priority research. Furthermore, it would also be highly desirable to investigate the potential effects of blocking these receptors on the disease severity in CD patients. Such studies could be undertaken in mice, which lack functional *KIR* genes but do carry their functional homologues.

Our findings provide not only important insights concerning the immunopathogenesis of CD, they may also lead to novel anti-CD therapeutics as well. It is noteworthy that *KIR* genes are expressed on the cell surface and hence are more accessible to antibodies or peptides than other target proteins that may be expressed inside cells, e.g., NOD2. KIR-specific antibodies, peptide mimics or soluble ligands, if developed, could be used for treating CD and related immune-mediated diseases.
